# Molecular Mechanisms of Plant Responses to Salt Stress

**DOI:** 10.3389/fpls.2022.934877

**Published:** 2022-06-27

**Authors:** Liang Ma, Xiaohong Liu, Wanjia Lv, Yongqing Yang

**Affiliations:** ^1^State Key Laboratory of Plant Physiology and Biochemistry, College of Biological Sciences, China Agricultural University, Beijing, China; ^2^Department of Art and Design, Taiyuan University, Taiyuan, China

**Keywords:** epigenetic regulation, hormonal regulation, salt stress, SOS pathway, signal transduction

## Abstract

Saline-alkali soils pose an increasingly serious global threat to plant growth and productivity. Much progress has been made in elucidating how plants adapt to salt stress by modulating ion homeostasis. Understanding the molecular mechanisms that affect salt tolerance and devising strategies to develop/breed salt-resilient crops have been the primary goals of plant salt stress signaling research over the past few decades. In this review, we reflect on recent major advances in our understanding of the cellular and physiological mechanisms underlying plant responses to salt stress, especially those involving temporally and spatially defined changes in signal perception, decoding, and transduction in specific organelles or cells.

## Introduction

Food production must increase by 70% worldwide by 2050 to meet the demands of the ever-increasing human population (High-Level Expert Forum, FAO, October 2009^1^). Thus, attaining food security and developing strategies to improve crop productivity and quality have become urgent aims. A major obstacle to improving crop productivity is soil salinization and alkalization, as salt stress severely restricts the germination rates, growth, development, and biomass accumulation of plants. Crop loss due to soil salinity poses an increasing threat to modern agriculture (Munns and Tester, 2008; Zhu, 2016; Yang and Guo, 2018a). In addition to rising levels of groundwater with high salt levels and increased evaporation due to drought, the increase in soil salinization is also caused by overirrigation and climate change (Rengasamy, 2006). Approximately one-fifth of irrigated lands worldwide are affected by soil salinization (Morton et al., 2019). Thus, the sustainable use of saline land resources and improving the agricultural output of saline soils are of paramount importance for global food security.

In contrast to halophytes, which can grow at high salt concentrations (>200 mM NaCl) (Munns and Tester, 2008; Flowers and Colmer, 2015), glycophytes are sensitive to high salinity. Most crops are glycophytes and are not suitable for growth in saline lands. An effective strategy for increasing crop yields in salinized agricultural lands is to design and breed salt-tolerant crop varieties. To achieve this goal, we must better understand (1) how to reduce soil salinity via phytoremediation or by improving agricultural practices; (2) the mechanisms by which high salinity leads to reduced water availability; (3) the toxic effects of sodium and chlorine ions on plants. Recent achievements in deciphering the underlying salt stress sensing and tolerance mechanisms in plants will greatly facilitate the breeding of salt-tolerant crop varieties. In this review, we briefly summarize recent progress in our understanding of salt stress sensing and signal transduction, focusing on how plants sense salt stress and transmit and decode salt stress signals to alter the gene expression and/or protein stability/activity in response to salt stress.

## Sensory Mechanisms of Salt Stress

**FIGURE 1:**
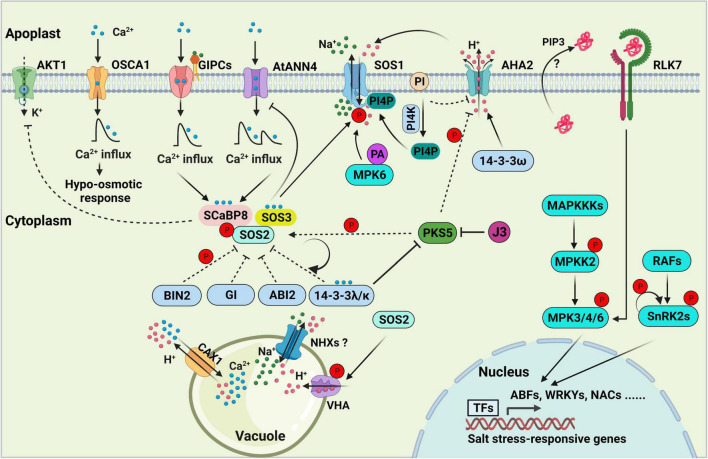
Salt stress signal transduction in plants. The SOS pathway, consisting of SOS3, SCaBP8, SOS2, and SOS1, is essential for decoding salt-induced calcium signals and maintaining ionic homeostasis in the plant cell. 14-3-3, GIGANTEA (GI), ABI2, and BIN2 proteins negatively regulate SOS pathway activity by directly interacting with SOS2 and repressing its kinase activity. PKS5-mediated phosphorylation of SOS2 enhances its interaction with 14-3-3, thereby inhibiting and maintaining basal levels of SOS2 activity under normal conditions. Arabidopsis K^+^ TRANSPORTER 1 (AKT1) activity is repressed by SCaBP8. GIPCs might function as monovalent-cation sensors that bind to Na^+^ and initiate calcium influx, which further activates the SOS pathway. AtANN4, a putative calcium-permeable transporter, might also generate calcium influx to activate the SOS pathway under salt stress. OSCA1 functions as an osmosensor to generate osmotic Ca^2+^ signaling in response to osmotic stress. Feedback regulation of AtANN4 by the SOS pathway is required to fine-tune the formation and duration of salt-induced calcium influx and long-term salt stress responses. Phosphatidylinositol (PI) directly binds to the C-terminus of the plasma membrane (PM) H^+^-ATPase AHA2 to repress its activity. PI is converted into phosphatidylinositol 4-phosphate (PI4P) to release the inhibition of AHA2 under salt stress. PI4P binds to and activates the PM Na^+^/H^+^ antiporter SOS1. PIP3 and RLK7 accumulate under salt stress, and the PIP3-RLK7 interaction contributes to the activation of RLK7, resulting in the activation of MPK3/6 to transduce stress signals. MAP kinase cascades are involved in regulating salt stress signal transduction. RAFs are required for the phosphorylation and activation of SnRK2s in response to salt-induced osmotic stress, and SnRK2 activity is amplified by auto-phosphorylation. In the nucleus, several specific transcription factors that are downstream targets of MPKs and SnRK2s bind to and activate the expression of salt stress-responsive genes. In the vacuole, NHXs, CAX1, the vacuolar Ca^2+^/H^+^ antiporter and vacuolar H^+^-ATPase (VHA) exclude Na^+^ from the cell. The dashed lines indicate regulatory roles under normal conditions.

The process of salt stress is divided into osmotic stress (early stage) and ionic toxicity (later stage) (Munns and Tester, 2008). To tolerate salt stress more effectively, plants have evolved various regulatory mechanisms that quickly perceive changes in Na^+^ concentrations and osmotic pressure caused by salt stress (Figure 1). NaCl or mannitol stimuli induce rapid increases in cytosolic Ca^2+^ levels within seconds (Knight et al., 1997), perhaps due to the strong coupling relationship between osmotic stress receptors and calcium channels. The increase in Ca^2+^ levels first occurs in roots (Tracy et al., 2008) and has been detected in several different cell types (Kiegle et al., 2000; Martí et al., 2013).

Exogenous expression of *Arabidopsis thaliana HISTIDINE KINASE 1* (*AtHK1*) suppressed the lethality of the temperature-sensitive and osmosensing-defective yeast mutation *sln1-ts* (Urao et al., 1999; Tran et al., 2007; Wohlbach et al., 2008), suggesting that AtHK1 functions in osmo-sensing. However, a subsequent study showed that the *hk1* mutant exhibits significant physiological responses to osmotic stress (Kumar et al., 2013), indicating that some other unknown proteins must be responsible for sensing osmotic stress. Based on these findings, Kurusu et al. (2013) proposed that hyper-osmotic stress is sensed by a mechanical Ca^2+^ channel, which was described as a mechano-osmotic sensory modality (Kurusu et al., 2013). The *osca1* (*reduced hyperosmolality-induced [Ca^2+^]_i_ increase 1*) mutant was isolated in *Arabidopsis* using calcium imaging-based unbiased forward genetic screening. Similar to the osmosensor TRPV4 in animals (Liedtke et al., 2000), OSCA1 is involved in osmotic stress-induced rapid signal transduction, intermediate cellular responses, and prolonged growth and development (Yuan et al., 2014). OSCA1 was identified as a plasma membrane hyperosmolality-gated calcium-permeable channel and a putative osmosensor required for osmotic stress-induced Ca^2+^ signatures (Yuan et al., 2014; Zhang et al., 2020).

Using the same strategy, a genetic screen for mutants impaired in intracellular calcium concentration ([Ca^2+^]_cyt_) increases specifically induced by sodium (Na^+^) was performed in *Arabidopsis*. This led to the identification of the *moca1* (*monocation induced Ca^2+^ increases 1*) mutant, which shows reduced cytosolic [Ca^2+^]_cyt_ spikes in response to salt treatment (Jiang et al., 2019). The increases in [Ca^2+^]_cyt_ in response to K^+^ or Li^+^ were also reduced in *moca1*, but it showed nearly wild type responses to H_2_O_2_, cold stress, and high external Ca^2+^ stimuli, indicating that *moca1* is mainly sensitive to monovalent cations. Furthermore, *moca1* only exhibits attenuated growth under Na^+^ stress, indicating that MOCA1 specifically regulates Na^+^ signaling (Jiang et al., 2019). MOCA1, an inositol phosphorylceramide glucuronosyltransferase also known as IPUT1, was also classified as PLANT GLYCOGENIN-LIKE STARCH INITIATION PROTEIN 6 (PGSIP6) in glucuronosyltransferase subfamily 8 (GT8) (Rennie et al., 2012, 2014). IPUT1 functions as an enzyme that transfers a glucuronic acid (GlcA) residue from UDP-GlcA to the precursor inositol phosphorylceramide (IPC) to form glycosyl inositol phosphorylceramide (GIPC) (Rennie et al., 2014). Indeed, *moca1* plants contained higher levels of IPCs but lower levels of GIPCs than wild type plants. It appears that GIPC directly binds Na^+^ on the cell surface, thereby preventing the subsequent depolarization of the cell membrane potential that gates Ca^2+^ channels (Jiang et al., 2019).

Based on the mechano-osmotic sensory modality, salt stress and osmotic stress have long been regarded as mediating the mechanical properties of the cell wall, which then senses and transduces the salt stress signal. Accumulating evidence suggests that the receptor-like kinase FERONIA (FER) (Feng et al., 2018), ANNEXINs (Laohavisit et al., 2013; Ma et al., 2019), plastid K^+^ exchange antiporters (KEAs) (Stephan et al., 2016), the mechanosensitive ion channel MscS-like (MSL) (Hamilton et al., 2015a,b), MID1-COMPLEMENTING ACTIVITY (MCA) (Kurusu et al., 2012a,b, 2013), and two-pore calcium channel family proteins (Choi et al., 2014) sense osmotic stress or salt stress by mediating salt-induced Ca^2+^ signaling or by perceiving salt stress-induced turgor or changes in cell structure.

Feronia functions as a sensor of cell wall softening and induce cell-specific calcium transients to maintain cell wall integrity under salt stress (Feng et al., 2018). Salt stress induces the processing and secretion of mature RAPID ALKALINIZATION FACTOR 22/23 (RALF22/23), which in turn interact with the cell wall-localized leucine-rich repeat extensins LRX3, LRX4, and LRX5, together with FER, to sense and transduce salt stress signals by monitoring cell wall integrity (Zhao C. et al., 2018). AtANN1 is an important regulator of calcium signaling and adaptive root growth that mediates reaction oxygen species (ROS)-induced increases in cytosolic calcium concentration ([Ca^2+^]_cyt_) under salt stress. The *atann1* mutant shows enhanced Na^+^ influx and K^+^ efflux at root epidermal cells and impaired root growth under saline conditions (Laohavisit et al., 2013). AtANN4, another calcium-dependent membrane-binding protein, and putative calcium-permeable transporter, is required for the salt stress response and salt-induced increases in [Ca^2+^]_cyt_ and is also essential for salt overly sensitive (SOS) pathway activation (Ma et al., 2019).

Pioneering studies demonstrated that KEA1 and KEA2 are targeted to the inner envelope membrane of the chloroplast, whereas KEA3 is targeted to the thylakoid membrane, and that these transporters are required for plasmid ion homeostasis and osmoregulation (Kunz et al., 2014; Stephan et al., 2016). Mutation of *KEA1*, *KEA2*, or *KEA3* leads to reduced rapid osmotic stress-induced calcium spikes, suggesting that Arabidopsis KEA1/2/3 function as osmotic sensors, endowing plants with the ability to sense the intensity of water limitation in response to osmotic stress (Stephan et al., 2016).

The rapidly reduced turgor pressure caused by high salinity is perceived by MSL and MCA family proteins. MSL10 is required for potentiating the [Ca^2+^]_cyt_ increase and ROS accumulation in response to cell swelling (Basu and Haswell, 2020), suggesting that MSL10 might function as a membrane-based sensor that perceives cell swelling. Pollen-localized MSL8, a membrane tension-gated ion channel, is required for the survival of pollen and full male fertility under hypo-osmotic shock due to rehydration (Hamilton et al., 2015a), and MSL2 and MSL3 have been implicated in osmotic homeostasis in chloroplasts (Haswell and Meyerowitz, 2006; Wilson et al., 2011; Veley et al., 2012). MCA1 is also required for cell wall integrity (Denness et al., 2011; Wormit et al., 2012) and for promoting Ca^2+^ influx upon mechanical stimulation (Nakagawa et al., 2007). Plasma membrane-localized OsMCA1 regulates Ca^2+^ influx and ROS generation in rice (*Oryza sativa*) under hypo-osmotic stress conditions (Kurusu et al., 2012a).

High salinity stress rapidly triggers H_2_O_2_ bursts in plant cells, which function as important stress signals (Wang W. et al., 2020). The leucine-rich-repeat (LRR) receptor kinase HYDROGEN-PEROXIDE-INDUCED CA^2+^ INCREASES1 (HPCA1) functions as a hydrogen peroxide sensor that perceives the stress-induced extracellular H_2_O_2_ burst and generates increased [Ca^2+^]_cyt_ under stress stimuli (Wu et al., 2020). The Arabidopsis Na^+^/H^+^ antiporter SOS1 may perform other activities in addition to its antiporter activity; its long cytoplasmic tail likely confers its salt stress sensing activity (Shi et al., 2002; Zhu, 2002). Plant harbors abundant proteins that can act as salt stress sensor(s) and sensing mechanisms operate in parallel will allow plants to decode the stress signals and adapt to salt stress more efficiently.

## Mechanisms of Osmotic and Ionic Signaling in Response to Salt Stress

During the long evolutionary process, plants have evolved a series of physiological, biochemical, and molecular regulatory mechanisms to respond to and resist salt stress (Figure 1), including the selective absorption, accumulation, or excretion of ions, the regionalization of Na^+^ in the cytoplasm through the membrane system, and the induction of stress tolerance gene expression (Roy et al., 2014; Zhu et al., 2016; Yang and Guo, 2018a; van Zelm et al., 2020). The cell surface-localized receptors rapidly sense external environmental stimuli (hypersaline stress), and second messengers such as Ca^2+^, ROS, and phytohormones are generated in a spatiotemporal-specific manner. These signals are decoded by diverse Ca^2+^-dependent proteins, including calmodulins (CaM), calmodulin-like proteins (CMLs), calcium-dependent protein kinases (CDPKs), CBL-interacting protein kinases (CIPKs)/SOS2-like protein kinases (PKSs) and calcineurin B-like proteins (CBLs)/SOS3-like calcium-binding proteins (SCaBPs) (Dodd et al., 2010). Finally, the Ca^2+^ signatures are translated into protein-protein interactions, protein phosphorylation/de-phosphorylation, phospholipid metabolism, or gene expression (Hashimoto and Kudla, 2011).

In Arabidopsis, the evolutionarily conserved SOS pathway is essential for plant adaptation to salt stress by exporting excess Na^+^ (Zhu, 2016; Yang and Guo, 2018a,b; van Zelm et al., 2020; Zhao et al., 2020). The classical SOS pathway includes three major components: the Na^+^/H^+^ antiporter SOS1; the serine/threonine-protein kinase SOS2, which harbors an N-terminal kinase domain similar to that of SUCROSE NON-FERMENTING 1 (SNF1)/AMPK; the helix E-loop-helix F hand (EF-hand) calcium-binding proteins SOS3 and SCaBP8/CBL10 (Zhu et al., 1998; Halfter et al., 2000; Liu et al., 2000; Shi et al., 2000; Qiu et al., 2002; Quan et al., 2007; Lin et al., 2009). The calcium sensors SOS3 and SCaBP8 perceive the salt-induced [Ca^2+^]_cyt_ to release the self-inhibition of SOS2 and promote its activity via interactions with its FISL motif (A, F, I, S, L, and F are absolutely conserved). These calcium sensors then recruit the activated SOS2 to the plasma membrane (Halfter et al., 2000; Guo et al., 2001; Quan et al., 2007). SOS3 primarily functions in roots, whereas SCaBP8 primarily functions in shoots in response to salt toxicity. The salt-hypersensitive phenotype of *sos3* was partially rescued by *ScaBP8* overexpression, but overexpressing *SOS3* failed to complement the salt-sensitive phenotype of *scabp8*, indicating that the functions of SOS3 and ScaBP8 are only partially redundant, and each plays additional and unique roles in plant responses to salt stress (Quan et al., 2007). ENDOSOMAL SORTING COMPLEX REQUIRED FOR TRANSPORT-I (ESCRT-I) subunit VPS23A VACUOLAR PROTEIN SORTING 23A (VPS23A) interacts with and assists in targeting SOS2 to the plasma membrane, which enhances the SOS2-SOS3 interaction on the plasma membrane (Lou et al., 2020). The plasma membrane-localized SOS2, with high levels of kinase activity, further phosphorylates and activates the Na^+^/H^+^ antiporter SOS1 (Quan et al., 2007; Lou et al., 2020).

Salt overly sensitive 1 (SOS1) is a key systemic determinant of Na^+^ extrusion from the cytosol to the apoplast, and its functional mutant *sos1-1* displays the exceptional sensitivity to salt stress (Shi et al., 2000, 2002; Qiu et al., 2002; Quintero et al., 2002, 2011). SOS1 forms a homodimer folded into an N-terminal transmembrane and a cytosolic, autoinhibitory C-terminal tail (Quintero et al., 2011; Núñez-Ramírez et al., 2012), which is activated when the amino acid residues (serine 1036 and serine 1038) at its C-terminus are phosphorylated by SOS2 (Quintero et al., 2011). Overexpression of SOS1 C terminus leads to increased salt tolerance by the sequestration of inhibitory 14-3-3 proteins (Duscha et al., 2020). SOS1 is also involved controlling long-distance Na^+^ transport from root to shoot (Shi et al., 2002; El Mahi et al., 2019). Besides SOS1, several Na^+^ transporters from different transporter protein families have been identified, for example, the HIGH-AFFINITY K^+^ TRANSPORTER 1 (HKT1) family transporter and the HAK-type Na^+^-selective ion transporter. HKT1 is responsible for promoting shoot Na^+^ exclusion by retrieving Na^+^ from xylem vessels (Ren et al., 2005; Møller et al., 2009). The AtHKT1 loss-of-function mutation was able to confer enhanced salt tolerance in both *sos1*, *sos2*, and *sos3* mutants by limiting the accumulation of cytosolic Na^+^ and mitigating the root-shoot Na^+^ translocation, indicating the interplays between HKT1 and SOS pathway is essential for modulating Na^+^ homeostasis in plant cells (Rus et al., 2001, 2004; Pabuayon et al., 2021). The HAK Na^+^ transporter is responsible for modulating root-to-shoot Na^+^ translocation and root Na^+^ content (Zhang et al., 2019; Wang et al., 2020). Salt stress-induced Ca^2+^ binds to ZmNSA1 and triggers its degradation, then promotes the transcription of Maize PM-H^+^-ATPases (MEAs) to enhance root H^+^ efflux, thus promoting the pump activity of SOS1 in maize (Cao et al., 2020). Numerous studies have revealed how plants adjust Na^+^ transporters and SOS pathway activities to balance plant growth and salt tolerance to facilitate adaptation to the ever-changing environment.

In the absence of salt stress, the kinase activity of SOS2 is inhibited by several regulators, including BRASSINOSTEROID-INSENSITIVE 2 (BIN2; Li et al., 2020), SOS2-LIKE PROTEIN KINASE 5 (PKS5; Yang Z. et al., 2019), 14-3-3 proteins (Zhou et al., 2014) and GIGANTEA (GI; Kim et al., 2013), all of which interact with SOS2 to repress its kinase activity. The protein phosphatase 2C ABA INSENSITIVE 2 (ABI2) interacts with SOS2 and might also negatively regulate its kinase activity (Ohta et al., 2003). The protein phosphatase interaction (PPI) motif in SOS2, a protein domain of 37 amino acid residues, is sufficient and necessary for the ABI2-SOS2 interaction (Ohta et al., 2003).

GIGANTEA and 14-3-3 proteins physically interact with SOS2 and suppress its kinase activity under normal conditions. Under salt stress conditions, GI and 14-3-3 are degraded by the 26S proteasome degradation pathway, resulting in the release of SOS2 from SOS2-GI/14-3-3 complexes and the activation of SOS2 (Kim et al., 2013; Zhou et al., 2014). 14-3-3 proteins are a family of conserved regulators that are considered to be phosphoserine binding proteins due to their ability to bind to numerous, functionally diverse phosphorylated signaling proteins, including kinases, phosphatases, and transmembrane receptors (reviewed in Fu et al., 2000). Phosphorylation of the amino acid residue (serine 294) in SOS2 enhanced its binding affinity to 14-3-3. PKS5 is responsible for phosphorylating SOS2 at this amino acid residue to promote the SOS2-14-3-3 interaction, thereby limiting SOS2 activity to basal levels in the absence of salt stress (Yang Z. et al., 2019). Salt stress represses the kinase activity of PKS5 by promoting the PKS5-14-3-3 interaction, thereby releasing the inhibition of SOS2 (Yang Z. et al., 2019).

The glycogen synthase kinase 3 (GSK3)-like kinase BIN2 phosphorylates and inhibits SOS2 activity to negatively regulate plant salt tolerance (Li et al., 2020). During the rapid recovery phase after salt stress, SOS3 and SCaBP8 promote the membrane distribution of BIN2, where BIN2 phosphorylates SOS2 at threonine 172 (T172) to represses its kinase activity. Meanwhile, downstream targets such as BES1 (BRI1-EMS-SUPPRESSOR 1) and BZR1 (BRASSINAZOLE RESISTANT1) are released to promote plant growth, indicating that BIN2 functions as a molecular switch between salt tolerance and growth recovery (Li et al., 2020).

Upon salt stress, not only are negative regulators of the salt stress response de-activated but positive regulators of this process are also activated. The key regulatory step in the activation of the SOS pathway is the amplification of SOS2 kinase activity in response to salt stress. As mentioned above, AtANN4 plays a critical role in mediating Ca^2+^ transients, which are essential for the activation of SOS2 kinase in Arabidopsis (Ma et al., 2019). In addition, the feedback regulation of AtANN4 fine-tunes the formation and duration of salt stress-induced Ca^2+^ transients, thereby optimizing SOS2 kinase activity in response to long-term salt stress (Ma et al., 2019). The salt-induced Ca^2+^ signature is decoded by 14-3-3 proteins, resulting in the increased repression of PKS5 activity, reduced SOS2^Ser294^ phosphorylation, and thus reduced repression of SOS2 activity by 14-3-3 proteins (Yang Z. et al., 2019). The SCaBP1/CBL2-PKS5 module and the calcium sensor SCaBP3/CBL7 all negatively regulate plasma membrane H^+^-ATPase activity under normal conditions, and PKS5 activity is repressed to release the H^+^-ATPase activity under salt stress (Fuglsang et al., 2007; Yang et al., 2010; Yang Y. et al., 2019). Together, these findings indicate that the salt-induced calcium signature is decoded by 14-3-3 and SOS3/SCaBP8 to activate or suppress SOS2 and PKS5 to further mediate plasma membrane Na^+^/H^+^ antiporter and H^+^-ATPase activity, respectively (Yang et al., 2010; Yang Z. et al., 2019). In the presence of salt stress, BIN2 dissociates from the plasma membrane, whereas SOS2 accumulates on the plasma membrane, also leading to the release of SOS2 inhibition and the activation of SOS1 (Quan et al., 2007; Li et al., 2020).

Besides the activation of the SOS pathway, plants have evolved an array of strategies that help them withstand salt stress, including the accumulation of protective metabolites, such as polyols, betaine, trehalsose, ectoine, proline, soluble sugars, polyamines (PAs), free unsaturated fatty acids, phosphatidylinositol, phosphatidic acid, and Late Embryogenesis Abundant (LEA) proteins, that buffer the negative effects of toxic ions (Hasegawa et al., 2000; Thole and Nielsen, 2008; Liu et al., 2015; Yang et al., 2021). Overexpression of *P5CS1*, encoding the rate-limiting proline biosynthesis enzyme Δ^1^-pyrroline-5-carboxylate synthetase 1, increased proline contents and osmotolerance in transgenic plants (Yoshiba et al., 1999; Székely et al., 2008; Szabados and Savouré, 2010). The plasma membrane-localized L-type amino acid transporter LAT1 (also known as PUT3) exhibits a polyamine transport activity (Fujita et al., 2012; Fujita and Shinozaki, 2014; Shen et al., 2016). SOS2 and SOS1 physically and genetically interact with PUT3 to modulate its polyamine transport activity in response to high salt conditions (Chai et al., 2020).

Free unsaturated fatty acids play an essential role in salt stress tolerance (Zhang et al., 2012) by activating the plasma membrane H^+^-ATPase by directly binding to its C-terminus (Han et al., 2017). Phosphatidylinositol (PI) directly binds to the C-terminus of the plasma membrane H^+^-ATPase AHA2 and inhibits its activity. PI is converted into phosphatidylinositol 4-phosphate (PI4P) under salt stress to mediate the removal of AHA2 inhibition, while the accumulated PI4P positively regulates salt tolerance by interacting with and activating SOS1 (Yang et al., 2021). NaCl treatment leads to increased PA levels and the increased production and enzymatic activity of phospholipase D (Yu et al., 2010; Wang et al., 2019). In turn, PA activates MITOGEN-ACTIVATED PROTEIN KINASE 6 (MPK6), which phosphorylates and activates SOS1 to improve plant salt tolerance (Yu et al., 2010).

Several other protein kinases are also involved in regulating salt stress responses. GEMINIVIRUS REP-INTERACTING KINASE 1 (GRIK1) and GRIK2 phosphorylate SOS2 at amino acid residue threonine 168 (T168), thereby increasing its kinase activity (Barajas-Lopez et al., 2018). The *grik1-2 grik2-1* mutant is sensitive to both glucose and high salt, indicating that GRIKs are not only involved in sugar/energy-sensing but also in salinity signaling pathways (Barajas-Lopez et al., 2018). CIPK8, another CIPK protein family member and a close homolog of SOS2, interacts with CBL10 and activates SOS1 to form the CBL10-CIPK8-SOS1 module, which extrudes excess Na^+^ (Yin et al., 2020). In addition, *OsMKK1* transcription and OsMKK1 kinase activity are markedly increased by salt treatment in rice. OsMKK1 then targets OsMPK4 to constitute a signaling pathway that positively regulates salt tolerance (Wang F. et al., 2014).

Arabidopsis MKK2 is activated by salt and cold stress, but not heat stress, hydrogen peroxide, or the flagellin-derived bacterial peptide elicitor flg22. Activated MKK2 specifically phosphorylates and activates MPK4 and MPK6, which induce the expression of cold- or salt stress-responsive genes (Teige et al., 2004). The *mkk2* mutant is hypersensitive to cold and salt stress, whereas plants overexpressing *MKK2* show increased salt and cold tolerance (Teige et al., 2004). MPK3/6 integrates cytokinin signaling by inducing the degradation of Arabidopsis RESPONSE REGULATOR 1 (ARR1), ARR10, and ARR12, thereby promoting salt tolerance (Yan et al., 2021).

By contrast, some MAP kinases play negative roles in salt stress tolerance. For example, the overexpression of *OsMAPK33* and *MKK9* led to increased salt sensitivity in rice and Arabidopsis, respectively (Xu et al., 2008; Lee et al., 2011). A T-DNA insertion mutant of *MKK9* displayed insensitivity to 150 mM NaCl during seed germination, along with increased expression of the stress-related genes *RESPONSIVE TO DEHYDRATION 22* (*RD22*) and *RD29* (Alzwiy and Morris, 2007). Moreover, the Arabidopsis *mapkkk20* knockout mutant showed enhanced tolerance to salt stress (Gao and Xiang, 2008), and the *mpk9 mpk12* double mutant showed reduced water loss compared to the wild type (Jammes et al., 2009). In addition, five MPK genes (*MPK9*, *MPK10*, *MPK11*, *MPK17*, and *MPK18*), two MKK genes (*MKK7* and *MKK9*), and four MEKK genes (MEKK3, *MEKK5*, *MEKK6*, and *MEKK7*) were induced by treatment with 200 mM NaCl (Moustafa et al., 2008), indicating that the MAP kinase signaling pathway plays a fundamental role in mediating plant salt tolerance. Raf-like protein kinases (RAFs) were classified as mitogen-activated protein kinase kinase kinases (MAPKKKs) in plants (Ichimura et al., 2002; Rao et al., 2010). Mutants of Raf-like kinase family members, such as *ctr1*, *raf10*, and *raf11*, are insensitive to ABA (Beaudoin et al., 2000; Lee et al., 2015), while the *raf5* and *sis8* mutants are hypersensitive to salt stress (Gao and Xiang, 2008). Plasma membrane-localized receptor-like kinases (RLKs) are also essential in sensing and transducing the salt stress signals (Li et al., 2014; Zhao X. et al., 2019; Zhou et al., 2022). The Lectin RLKs (LecRLKs) is reported to play critical roles in mediating plant salt stress and ABA responses (Vaid et al., 2013). The SALT INTOLERANCE 1 (SIT1) encodes a putative LecRLK and mediates salt sensitivity by activating MPK3/6 and promoting ethylene production and ROS accumulation in rice (Li et al., 2014). The salt-induced kinase activity of SIT1 is constrained by PP2A regulatory subunit B’κ-mediated dephosphorylation in its activation loop (Li et al., 2014; Zhao J. et al., 2019). In addition, the phosphorylation of RECEPTOR-LIKE KINASE 7 (RLK7) is enhanced with NaCl treatment, which positively regulates salt stress response by activating downstream MPK3/6 in Arabidopsis (Zhou et al., 2022). Understanding the specific mechanisms of salt stress signal transduction is essential for exploiting the potential of molecular and genetic markers/tools to create/breed salt-resilient crops.

## Epigenetic Regulation

Epigenetic regulation, including DNA methylation, histone modifications, histone variants, and some non-coding RNAs, plays essential roles in regulating plant adaptation to abiotic stress (Kinoshita and Seki, 2014; Chang et al., 2020; Wu et al., 2022). HKT1 is a salt tolerance determinant that mediates Na^+^ entry and high-affinity K^+^ uptake in roots (Rus et al., 2001). A putative small RNA target region of the *HKT1* promoter is heavily methylated. The RNA-directed DNA Methylation (RdDM) component RDR2 is responsible for the methylation and expression of *HKT1*, indicating that the RdDM pathway functions in the salt stress response by negatively regulating *HKT1* expression (Baek et al., 2011; Kumar et al., 2017). RDM16, another component of the RdDM pathway, regulates the overall methylation of transposable elements and the regions surrounding genes by influencing Pol V transcript levels (Huang et al., 2013). The *rdm16* mutant is hypersensitive to salt stress and ABA, pointing to a tight connection between salt stress responses and DNA methylation (Huang et al., 2013).

In addition to DNA methylation, changes in histone modifications are also involved in regulating salt stress tolerance (Kim et al., 2015). Several ABA- and salt stress-responsive genes showed increased histone H3K9K14 acetylation and H3K4 trimethylation but decreased H3K9 dimethylation after ABA or salt treatment, indicating that the expression of stress-responsive genes is associated with changes in histone modifications (Chen et al., 2010). A higher-order mutant of *HISTONE DEACETYLASE 6* (*HDA6*) showed decreased expression of ABA- and abiotic stress-responsive genes, thereby showing a salt-hypersensitive phenotype (Chen et al., 2010). The histone deacetylase HD2C interacts with HDA6 to repress the expression of *ABI1* and *ABI2* via histone modifications, and the *hd2c* mutant also showed increased sensitivity to ABA and salt stress (Luo et al., 2012).

The floral initiator SKB1 (SHK1 KINASE BINDING PROTEIN1) perceives salt stress and disassociates with chromatin to decrease H4R3sme2 (symmetric dimethylation of histone4 arginine3) levels to induce the transcription of *FLOWERING LOCUS C*, thereby regulating flowering time in Arabidopsis under salt stress (Zhang et al., 2011). Both transcriptomic changes and alternative mRNA splicing play vital roles in regulating the salt stress response (Barbazuk et al., 2008; Kornblihtt et al., 2013; Kong et al., 2014). The expression of linker histone variants is dependent on indicated environmental stimuli, for example, *H1.S* of tomato (Scippa et al., 2004) and *HIS1-3* of Arabidopsis (Ascenzi and Gantt, 1997) could be induced by drought stress, and ABA treatment. However, the expression of *HIS1-3* could be inhibited by salt stress (Wu et al., 2022). HIS1-3 negatively regulates plant salt stress response through the SOS pathway and the higher-order mutant of HIS1-3 confers plants’ enhanced salt tolerance in Arabidopsis (Wu et al., 2022). Arabidopsis SERRATE (SE) interacts with HYPONASTIC LEAVES 1 (HYL1) and CHR2 to facilitate microRNA biogenesis and fine-tune primary-microRNA processing (Lobbes et al., 2006; Yang et al., 2006; Wang Z. et al., 2018). High salinity represses the expression of SE at both the mRNA and protein levels, and the *se* mutant is hypersensitive to salt stress (Mou et al., 2021). SE positively regulates plant salt stress tolerance by modulating the pre-mRNA splicing of salt stress-responsive genes (Mou et al., 2021).

A study comparing the small RNA (sRNA) transcriptomes of the mangroves *Bruguiera gymnorrhiza* and *Kandelia candel* found that mangroves exhibit distinct sRNA expression patterns and regulatory networks that differ from those of glycophytes, indicating that changes in sRNA expression and sRNA-regulatory networks during evolution are essential for salt stress adaptation in plants (Wen et al., 2016). Salt stress decreases the accumulation of 24-nt siRNAs in Arabidopsis, thereby altering the expression of the transcription factor gene *AtMYB74* to help the plant adapt to high salinity conditions (Xu et al., 2015). Indeed, overexpressing *AtMYB47* led to hypersensitivity to salt stress during seed germination (Xu et al., 2015).

## Phytohormone Signaling Pathways

**FIGURE 2:**
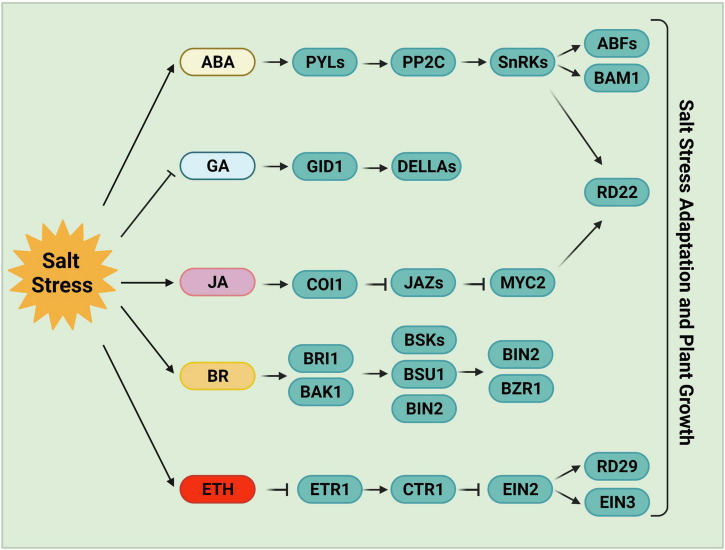
Phytohormone-mediated salt stress responses. ABA, one of the most important stress response hormones, plays a crucial role in salt stress tolerance. ABA-activated SnRK2s regulate stomatal closure, osmotic homeostasis, and gene expression. Salt stress negatively regulates the accumulation of bioactive GAs, and the reduced GAs levels or inactivated GAs promote plant salt tolerance following germination. JA levels increase and JA signaling is activated by high salinity stress. JA is required for the inhibition of primary root growth, which may be an adaptive strategy for survival under salt stress. BR, a growth-promoting phytohormone, accumulates upon salt stress to positively regulate plant salt tolerance. BR induces the formation of the BRI1-BAK1 heterodimer, which then initiates the phosphorylation relay cascades among BSKs, BSU1, and BIN2, ultimately remodeling gene expression via the regulation of BZR1 and BES1. Ethylene also accumulates under salt stress in plants. The components involved in ethylene homeostasis or the ethylene signaling pathway play either positive or negative roles in salt stress responses. ABA, abscisic acid; GA, gibberellin; JA, jasmonic acid; BR, brassinosteroid; ETH, ethylene.

Phytohormones, including ABA, jasmonic acid (JA), gibberellin (GA), brassinosteroids (BRs), and ethylene are essential for plant growth and development and mediate biochemical and physiological responses to environmental stress (Figure 2), such as osmotic, salt, drought, cold and pathogen stress (Peleg and Blumwald, 2011; Yu et al., 2020). ABA, a 15-carbon sesquiterpenoid, plays important role in regulating plant growth, osmolyte accumulation, stomatal closure, leaf senescence, and root growth under abiotic stress. ABA also acts as an endogenous messenger involved in salt and drought stress signal transduction to initiate downstream gene expression (Finkelstein, 2013; Zhu, 2016).

The expression of ABA biosynthetic genes is strongly induced in certain tissues in response to salinity or water deficit, including ABA DEFICIENT genes (ABAs), *ALDEHYDE OXIDASE 3*, and NINE-*CIS*-EPOXYCAROTENOID DIOXYGENASE genes (NCEDs) (Barrero et al., 2006; Endo et al., 2008). *NCED3*, encoding a key enzyme for ABA biosynthesis, is induced in leaves under water-deficient stress (Iuchi et al., 2001; Tan et al., 2003; Endo et al., 2008). *NCED5*, which encodes a rate-limiting enzyme in ABA biosynthesis, is also rapidly induced under salt stress (Huang et al., 2019). The root-derived CLE25 (CLAVATA3/EMBRYO-SURROUNDING REGION-RELATED 25) protein transmits water-deficiency signals from the vascular system in roots to leaves, where it is sensed by BAM (BARELY ANY MERISTEM), indicating that the CLE-BAM module functions as a long-distance signaling pathway in response to dehydration stress (Takahashi et al., 2018). The application of CLE25 to roots induces *NCED3* expression and the accumulation of ABA in leaves, leading to a level of stomatal closure similar to that induced by ABA application. Consistently, the *CLE25* CRISPR-Cas9-derived knockout mutant *cle25* and the *bam1-5 bam3-3* double mutant are hypersensitive to both dehydration and salinity stress (Takahashi et al., 2018).

A very rapid and massive increase in ABA levels is observed in both roots and shoots under stress conditions (Jia et al., 2002; Fricke et al., 2004). This increase is sensed by the PYRABACTIN RESISTANCE 1 (PYR1)/PYR1-like (PYL)/REGULATORY COMPONENT OF ABA RECEPTORS (RCAR) family (PYLs) receptors (Ma et al., 2009; Park et al., 2009; Vlad et al., 2009). Once the PYLs bind to ABA, their conformation changes, allowing them to interact with protein phosphatase 2Cs (PP2Cs) and inhibit their activities, thus releasing SNF1-RELATED PROTEIN KINASE 2s (SnRK2s) from repression (Ma et al., 2009; Park et al., 2009). The released SnRK2s phosphorylate multiple downstream anion efflux channels and transcription factors, leading to decreased turgor pressure, stomatal closure, and gene expression reprogramming (Uno et al., 2000; Mustilli et al., 2002; Furihata et al., 2006; Fujii et al., 2007; Negi et al., 2008; Chen K. et al., 2020; Lin et al., 2020; Takahashi et al., 2020).

All 10 members of the SnRK2 family except SnRK2.9 are activated by osmotic stress (Boudsocq et al., 2004; Zhu, 2016), and the *snrk2.1/2/3/4/5/6/7/8/9/10* decuple mutant is hypersensitive to osmotic stress (Fujii et al., 2011). The activation of SnRK2s induced by ABA (but not osmotic stress) is abolished in *ABA insensitive 1* (*abi1-1*) or in higher-order mutants of PYR/PYL/RCAR ABA receptors (Vlad et al., 2010; Zhao Y. et al., 2018). Raf-like kinases (RAFs, especially B2, B3, and B4 RAFs) are required for the phosphorylation and activation of SnRK2s in response to early osmotic stress (Lin et al., 2020). The activated SnRK2s then *trans*-phosphorylate other SnRK2s to amplify the response (Lin et al., 2021). Higher-order mutants of *RAFs* are hypersensitive to osmotic stress induced by mannitol, NaCl, or polyethylene glycol treatment (Lin et al., 2020).

Abscisic acid-activated SnRK2s are also involved in regulating starch content in response to salt stress. SnRK2s phosphorylate and activate AREB/ABF transcription factors, which bind to the promoters of *β-AMYLASE1* (*BAM1*) and *α-AMYLASE3* (*AMY3*) and activate their expression (Thalmann et al., 2016). BAM1 and AMY3 mediate the degradation of starch to release sugar and sugar-derived osmolytes (Thalmann et al., 2016). The Arabidopsis *amy3 bam1* double mutant is hypersensitive to osmotic and salinity stress (Thalmann et al., 2016). When environmental stress subsides or in the absence of stress conditions, the target of rapamycin (TOR) kinase phosphorylates PYLs to negative regulate their activity, resulting in the disassociation of the PYL-ABA-PP2C complex and the inactivation of ABA and stress signaling, which is sufficient to promote growth recovery (Wang P. et al., 2018). ABI2 is involved in modulating salt tolerance by suppressing the SOS pathway via the ABI2-SOS2 interaction (Ohta et al., 2003).

Emerging evidence points to the coordination between the JA pathway and salt stress signal transduction (Kazan, 2015; Delgado et al., 2021). Analysis of the root transcriptome of sweet potato revealed the upregulation of JA-biosynthesis genes under salt stress; the intensity of upregulation was much greater in a salt-tolerant variety vs. a salt-sensitive line. The JA signaling pathway is also involved in regulating the expression of salt stress-responsive genes. Therefore, the JA signaling pathway is essential for salt tolerance (Ma et al., 2006; Wang J. et al., 2020). A high-order mutant of *LIPOXYGENASE3* (*LOX3*) exhibited salt hypersensitivity, which was rescued by treatment with methyl jasmonate, indicating that JA plays a positive role in salt tolerance (Ding et al., 2016).

The transcription factor MYC2, the regulatory hub of the JA pathway, also plays an essential role in salt tolerance (Abe et al., 2003; Chini et al., 2016; Valenzuela et al., 2016; Zander et al., 2020). MYC2 binds to the promoter of *RD22* and activates its expression in response to NaCl or ABA treatment (Iwasaki et al., 1995; Abe et al., 2003). However, the salt stress-mediated activation of the JA signaling pathway inhibits cell elongation in the root elongation zone, and JA-related mutants (*aos, col1, jaz3, myc2/3/4*) exhibit longer primary roots than wild type plants under salt stress (Valenzuela et al., 2016). Finally, RICE SALT SENSITIVE3 (RSS3) interacts with Class-C bHLH transcription factors and JASMONATE ZIM-DOMAIN (JAZ) proteins (which negatively regulate JA signaling) to promote root cell elongation in rice in response to salt stress (Toda et al., 2013). Overexpressing *OsJAZ9* and *OsJAZ8* increased plant tolerance to soil salinity (Wu et al., 2015; Peethambaran et al., 2018). Therefore, the JA signaling pathway is required to inhibit root growth under high salinity conditions. Together, these findings suggest that JA has dual functions in plant responses to salt stress.

The phytohormone GA also plays an essential role in regulating plant growth under salt stress (Khan et al., 2012; Colebrook et al., 2014). Several GA-metabolism-related proteins positively regulate the salt stress response. Conversely, salt stress usually reduces bioactive GA levels and increases the accumulation of DELLA proteins, resulting in dwarfism and enhanced stress tolerance (Achard et al., 2006; Qin et al., 2011). For instance, microarray data indicate that several genes encoding GA deactivation enzymes are upregulated by high salinity, including *GA2-oxidase 1* (*GA2ox1*), *GA2ox2*, *GA2ox4*, *GA2ox6*, and *GA2ox8* (Kilian et al., 2007; Magome et al., 2008). *GA2ox7*, encoding a C20-GA deactivation enzyme, is also strongly induced by salt stress. A higher-order *GA2ox* mutant showed higher sensitivity to salt stress than the wild type (Magome et al., 2008). Consistently, a quadruple-DELLA mutant lacking GAI, RGA, RGL1, and RGL2 (Cheng et al., 2004) shows less salt-triggered inhibition of root growth and flowering than the wild type but is hypersensitive to salt-induced death (Achard et al., 2006), indicating that DELLA proteins play a central role in the trade-off between growth limitation and survival under salt stress.

Brassinosteroids are plant growth-promoting steroid hormones that play critical roles in plant growth, development, and stress responses (Nolan et al., 2019; Planas-Riverola et al., 2019). BR signaling is essential for plant salt tolerance (Cui et al., 2012; Zhao X. et al., 2019). BRs such as 24-epibrassinolide (eBL) binds to the receptor BR INSENSITIVE1 (BRI1) (Friedrichsen et al., 2000; He et al., 2000) or its homologs BRI1-LIKEs (BRLs) (Caño-Delgado et al., 2004; Kinoshita et al., 2005) and the coreceptor BAK1 to initiate the BR signaling pathway, leading to a phosphorylation relay cascade among BAK1, BRASSINOSTEROID-SIGNALING KINASEs (BSKs), BRI1 SUPPRESSOR 1 (BSU1) and BRASSINOSTEROID-INSENSITIVE 2 (BIN2) (Li and Nam, 2002; Russinova et al., 2004; Hohmann et al., 2018). BIN2 activity is inhibited by the activated BSU1, thereby promoting BR-induced gene expression and inhibiting BR-repressed gene expression via the regulation of the transcription factors BZR1, BES1, and other transcription factors or cofactors (Nolan et al., 2019; Planas-Riverola et al., 2019).

Under high salinity, the activity of the ethylene biosynthesis enzyme ACS (1-aminocyclopropane-1-carboxylate synthase) is enhanced by BR pretreatment, resulting in ethylene accumulation and better adaptation to salt stress (Tao et al., 2015; Zhu et al., 2016). Conversely, shutting down ethylene production represses BR-induced antioxidant enzyme activity and decreases salt tolerance (Tao et al., 2015; Zhu et al., 2016). High salinity strongly inhibits roots growth due to reduced accumulation of BZR1 in the nucleus and the repression of BR signaling (Geng et al., 2013). However, exogenous BR application partially rescued salt-induced growth inhibition (Zeng et al., 2010; Liu et al., 2014; Zhu et al., 2016). OsSERK2 localizes to the plasma membrane in rice and interacts with the BR receptor OsBRI1 to facilitate BR signaling. Notably, the CRISPR/Cas9 edited *osserk2* mutant is impaired in BR signaling and shows hypersensitivity to salt stress (Dong et al., 2020). Finally, BIN2 is a negative regulator of the SOS pathway, which functions partially independently of the BR signaling pathway to balance plant growth and stress responses (Li et al., 2020). All of these findings demonstrate that the sensing and signaling of phytohormone pathways contribute to salt stress tolerance.

## Salt Stress Responses in Various Organelles

The plant cell wall consists of cellulose, pectins, hemicellulose, and various glycoproteins that help modulate cell wall extensibility. This property determines cell shape and size via the mechanical control of cell enlargement and expansion, thereby governing tissue and organ morphology (Cosgrove, 2005; Le Gall et al., 2015). Accumulating evidence demonstrates the important roles of the cell wall in plant responses to abiotic stress (Le Gall et al., 2015; Reviewed in Endler et al., 2015). The cell wall is a sensor of salt stress, and salt stress perception-to-signaling cascades mainly function at the cell wall-plasma membrane interface (Kacperska, 2004; Hou et al., 2005; Feng et al., 2018; Zhao C. et al., 2018). The nuclear-localized Agenet domain-containing protein SWO1 (SWOLLEN 1) functions together with importin α IMPA1 and IMPA2 to maintain cell integrity in Arabidopsis under salt stress (Wang et al., 2021). Several receptor-like protein kinases (RLKs) are regarded as sensors that perceive salt stress signals, such as the wall-associated kinases (WAKs) and FER (Baluska et al., 2005; Hou et al., 2005; Kohorn and Kohorn, 2012; Feng et al., 2018). *WAK-LIKE KINASE 4* (*WAKL4*) but not *WAK1* is highly induced under high salinity conditions (Hou et al., 2005), suggesting that WAKs play different roles in response to different stress conditions. The receptor-like kinase FER binds to RALF, senses salt-induced cell wall damage, and transduces the signals to downstream targets to maintain cell wall integrity under salt stress (Feng et al., 2018). The cell wall leucine-rich repeat extensins (LRX) 3/4/5 function together with the FER-RALF module to transduce cell wall signals to mediate plant growth and salt tolerance (Zhao C. et al., 2018).

In addition to sensing salt stress signals, the cell wall also plays a significant role in protecting the cell from salt stress-induced ionic toxicity. Several mutants with defective cell wall integrity are hypersensitive to salt. For instance, mutants of *SOS6*, encoding the cellulose synthase-like protein AtCSLD5, show reduced levels of arabinose, rhamnose, and galacturonic acid, which normally confer salt/osmotic stress tolerance. Thus, the *sos6* mutant exhibits elevated ROS contents and a salt-hypersensitive phenotype under salt stress (Zhu et al., 2010). Membrane-localized cellulose synthase (CesA) complexes are required to synthesize cellulose, the main component of the cell wall (McFarlane et al., 2014). CESA1, CESA3, and CESA6 are required for primary cell wall synthesis (Arioli et al., 1998; Fagard et al., 2000), while CESA4, CESA6, and CESA8 are required for secondary cell wall synthesis (Taylor et al., 2003; Endler and Persson, 2011). The sustained cellulose synthesis conferred by CESA1, CESA6, and CELLULOSE SYNTHASE-INTERACTIVE PROTEIN 1 (CSI1) is important for salt tolerance in Arabidopsis (Kang et al., 2008; Gu et al., 2010; Li et al., 2012; Zhang et al., 2016), as knocking out either *CESA1*, *CESA6* or *CSI1* conferred increased sensitivity to salt stress. Two plant-specific components of the cellulose synthase complex, CC1 (COMPANION OF CELLULOSE SYNTHASE 1) and CC2 interact with CesA proteins and microtubules to promote the CesA activity and microtubule dynamics required for hypocotyl growth under salt stress (Endler et al., 2015).

The endoplasmic reticulum (ER) is the main site for the modification or folding of secretory and membrane proteins to achieve their native structures. In addition, the ER is a fundamental organelle involved in signal transduction that allows plants to adapt to diverse environmental stresses (Vitale and Boston, 2008; Liu and Howell, 2010; Jin and Daniell, 2014). The accumulation of unfolded or misfolded proteins is induced by various biotic and abiotic stresses, ER stress (Liu et al., 2007; Ye et al., 2011; Zhang and Wang, 2012). The cell has developed several important strategies to alleviate ER stress, including the accelerated degradation of misfolded proteins through the ER-associated degradation (ERAD) pathway (Vembar and Brodsky, 2008; Chen Q. et al., 2020). Numerous misfolded proteins accumulate in the ER under salt stress, which is recognized by the ERAD pathway and ubiquitinated and degraded through the ubiquitin/26S proteasome system (Liu et al., 2011), indicating that the ERAD pathway is essential for plant survival under high salinity conditions.

Plants also alleviate salt stress by activating the expression of ER chaperones. HRD3A, the functional homolog of the yeast HRD1/HRD3 complex, is an active component of the ERAD complex in Arabidopsis. The *hrd3a* mutant exhibits an enhanced unfolded protein response under ER stress and increased sensitivity to salt stress (Liu et al., 2011). Accumulating evidence indicates that the ERAD complex plays a positive role in the salt stress response. For example, the ERAD complex mutants *mns4*/*mns5*, *atos9*, and *hrd1a*/*hrd1b* are hypersensitive to salt stress (Hüttner et al., 2012, 2014; Su et al., 2012). In addition, a defect in UBC32, a salt stress-induced functional ubiquitin conjugation enzyme (E2) required for the activation of the ERAD complex in Arabidopsis, confers increased tolerance to salt stress via a BR-dependent pathway (Cui et al., 2012). SES1 (SENSITIVE TO SALT 1), an ER-localized chaperone, protects plants from salt stress by alleviating salt-induced ER stress. The salt-sensitive phenotype of *ses1* is due to the over-activation of the unfolded protein response. In addition, the ER stress sensor bZIP17 directly binds to the promoter of *SES1* to activate its transcription under salt stress (Guan et al., 2018). Finally, a recent study demonstrated that the Arabidopsis receptor-like kinase SIMP1 (SALT-INDUCED MALECTIN-LIKE DOMAIN-CONTAINING PROTEIN 1) positively modulates plant salt tolerance by interacting with and stabilizing the putative proteasome maturation factor UMP1A, thus leading to enhanced proteasome maturation and ERAD efficiency, resulting in the mitigation of salt stress-induced ER stress (He et al., 2021).

## Regulation of Crosstalk Between Salt Stress and Other Signaling Pathways

Accumulating evidence points to a tight connection between the salt signaling pathway and other signal transduction pathways. For instance, light signaling plays a vital role in shaping the salt stress response. The localization of CONSTITUTIVE PHOTOMORPHOGENIC1 (COP1) is tightly controlled by light signals (von Arnim and Deng, 1994), whereas salt treatment promotes the translocation of COP1 to the cytosol (Yu et al., 2016). Light signaling is involved in regulating the salt-induced transcriptional memory response of *P5CS1* to proline (Feng et al., 2016). In addition, PHYTOCHROME-INTERACTING FACTOR4 (PIF4), a negative regulator of photomorphogenesis, negatively regulates plant salt tolerance by directly modulating the expression of diverse salt-responsive genes (Leivar and Quail, 2011; Lee and Choi, 2017; Sakuraba et al., 2017). Moreover, the regulation of the salinity stress response depends on diurnal cycles and the circadian clock via modulating the expression of *RD29A* and *SOS1*. The abundance of SOS1 protein also appears to occur in a diurnal cycle (Park et al., 2016). Finally, low levels of NaCl in the soil severely inhibit shade-induced hypocotyl elongation via the BR and ABA signaling pathways (Hayes et al., 2019).

Genetic and molecular studies have implicated pattern recognition receptors (PRRs) in salt stress tolerance. Arabidopsis PROPEP3 functions in the plant immune system (Huffaker et al., 2006) and positively regulates the salt stress response (Nakaminami et al., 2018). *PROPEP3* is highly induced under salinity stress conditions, and both *PROPEP3* overexpression and Pep3 application enhance salt stress tolerance via PEP-RECEPTOR l (Nakaminami et al., 2018). Similarly, PAMP-INDUCED SECRETED PEPTIDE 3 (PIP3) functions together with RLK7, a leucine-rich repeat receptor-like kinase (LRR-RLK), to further activate MPK3/6, therefore conferring salt tolerance (Zhou et al., 2022). All of these findings suggest that the sensing and signaling of damage-associated molecular patterns contribute to salt stress tolerance.

## Future Perspectives

Soil salinity severely threatens plant growth, crop productivity, and food security worldwide (Yang and Guo, 2018a,b; van Zelm et al., 2020). Identifying and characterizing the determinants and regulatory mechanisms of salt stress signaling represents the most effective way to breed salt-tolerant crops and improve agricultural development. In the last two decades, many new advances have been made in elucidating the key components of the salt stress response. In addition, several genetic loci involved in salt tolerance have been identified and the underlying genes cloned, representing candidate targets for designing the next generation of crop varieties. Studies of the SOS pathway have clearly demonstrated the mechanism of sodium ion (Na^+^) efflux and ion homeostasis. However, continuous efforts leading to substantial new discoveries are needed to better understand and further improve salt tolerance in plants.

Although GIPCs were characterized as potential monovalent-cation sensors (Jiang et al., 2019), the identification of other sodium sensors or receptors is still the most important goal of plant salt stress signaling research. As salt stress severely inhibits or destroys chloroplast development and photosynthesis, it is important to investigate whether salt sensors are present in different organelles, such as the chloroplast, ER, and vacuole. In addition, salt stress induces rapid calcium signaling in the cytosol (Knight et al., 1997; Zhu et al., 2016; Ma et al., 2019), and the long-distance transmission of Ca^2+^ waves from root to shoot is channeled through the cortex and endodermal cell layers, which is dependent on the vacuolar ion channel TPC1 (Choi et al., 2014). Therefore, it is critical to understand how the local salt stress signal in roots is sensed by different tissues and how plants integrate tissue-specific signals to confer stress tolerance throughout the plant.

The evolutionarily conserved SOS pathway primarily mediates Na^+^ homeostasis through the activation of the SOS3/SCaBP8-SOS2-SOS1 module under high salinity stress. Higher-order mutants of *SOS2* and *SOS1* exhibit severely reduced primary and lateral root growth under salt stress. As root system architecture is not only shaped by salt stress signals but also by plant nutrient status, whether and how the SOS pathway integrates different signal pathways (such as nutrient signaling and salt stress signaling) to balance plant root growth and stress tolerance needs to be explored in the future.

## Author Contributions

LM wrote the manuscript. XL and WL participated in writing and modification of the manuscript. LM and YY edited the manuscript. All authors have read and agreed to the published version of the manuscript.

## Conflict of Interest

The authors declare that the research was conducted in the absence of any commercial or financial relationships that could be construed as a potential conflict of interest.

## Publisher’s Note

All claims expressed in this article are solely those of the authors and do not necessarily represent those of their affiliated organizations, or those of the publisher, the editors and the reviewers. Any product that may be evaluated in this article, or claim that may be made by its manufacturer, is not guaranteed or endorsed by the publisher.

## Footnotes

1
http://www.fao.org
